# (Pro)renin receptor contributes to renal mitochondria dysfunction, apoptosis and fibrosis in diabetic mice

**DOI:** 10.1038/s41598-019-47055-1

**Published:** 2019-08-12

**Authors:** Caixia Li, Luis C. Matavelli, Safia Akhtar, Helmy M. Siragy

**Affiliations:** Division of Endocrinology and Metabolism, University of Virginia Health System, Charlottesville, VA 22903 USA

**Keywords:** Molecular biology, Kidney

## Abstract

Recently we demonstrated that increased renal (Pro)renin receptor (PRR) expression in diabetes contributes to development of diabetic kidney disease. However, the exact mechanisms involving PRR activity and diabetic kidney dysfunction are unknown. We hypothesized that PRR is localized in renal mitochondria and contributes to renal fibrosis and apoptosis through oxidative stress-induced mitochondria dysfunction. Controls and streptozotocin-induced diabetic C57BL/6 mice were injected with scramble shRNA and PRR shRNA and followed for a period of eight weeks. At the end of study, diabetic mice showed increased expressions of PRR and NOX4 in both total kidney tissue and renal mitochondria fraction. In addition, renal mitochondria of diabetic mice showed reduced protein expression and activity of SOD2 and ATP production and increased UCP2 expression. In diabetic kidney, there was upregulation in the expressions of caspase3, phos-Foxo3a, phos-NF-κB, fibronectin, and collagen IV and reduced expressions of Sirt1 and total-FOXO3a. Renal immunostaining revealed increased deposition of PRR, collagen and fibronectin in diabetic kidney. In diabetic mice, PRR knockdown decreased urine albumin to creatinine ratio and the renal expressions of PRR, NOX4, UCP2, caspase3, phos-FOXO3a, phos-NF-κB, collagen, and fibronectin, while increased the renal mitochondria expression and activity of SOD2, ATP production, and the renal expressions of Sirt1 and total-FOXO3a. In conclusion, increased expression of PRR localized in renal mitochondria and diabetic kidney induced mitochondria dysfunction, and enhanced renal apoptosis and fibrosis in diabetes by upregulation of mitochondria NOX4/SOD2/UCP2 signaling pathway.

## Introduction

(Pro)renin receptor (PRR) is a single transmembrane domain that is located in cell membrane and in some cell organelles such as endoplasmic reticulum and Golgi apparatus. PRR has been considered a component of the renin-angiotensin-system through its direct binding to renin or prorenin^1,2^. In addition, this receptor also presents angiotensin-independent actions by directly stimulating a variety of intracellular signal pathways, leading to cellular inflammation, apoptosis, fibrosis, proliferation and autophagy^3–5^. Our previous studies demonstrated that PRR expression is markedly increased in diabetic kidney^6,7^ and that upregulation of PRR contributed to high glucose induced mouse podocytes damage through PI3K/AKT/mTOR and Wnt3a/beta-catenin/snail signaling pathway^4,8^. In mesangial cells and streptozocin-induced diabetes rat model, increased renal PRR expression contributed to development of diabetic kidney disease via enhanced TGF-β1-connective tissue growth factor signaling pathway^9^. In contrast, downregulation of PRR attenuated hyperglycemia-induced renal damage and high glucose-associated cell dysfunction^9^.

Mitochondria is involved in most cell oxidative stress reactions and ATP production. Previous studies showed increased renal oxidative stress and high level accumulation of reactive oxygen species (ROS) in diabetic kidney^10,11^. This pathological process involves increased mitochondria NADPH oxidase 4 (NOX4) levels and reduced expression and activity of superoxide dismutase (SOD)^12–14^. Increased rate of renal ROS production in diabetes can cause damage to the mitochondria membrane and DNA and increase the expression of uncoupling proteins 2 (UCP2)^15–17^, leading to increased proton leakage across the outer mitochondria membrane to the inner membrane. This process reduces the mitochondria membrane potential resulting in decreased ATP production^18–21^.

The localization and functioning of PRR in mitochondria are not well established. Recent studies showed that PRR increased hydrogen peroxide production in collecting duct cells^22^ and promoted fibrosis in human embryonic kidney cells^23^ through a NOX4-dependent mechanism. However, it is unclear if hyperglycemia-induced PRR overexpression is linked to mitochondria derived oxidative stress and dysfunction.

In this study, we hypothesized that hyperglycemia increased PRR expression in diabetic kidney and in renal mitochondria fractions leading to increased renal oxidative stress, fibrosis and apoptosis. We evaluated the influence of upregulated renal PRR expression on mitochondria derived oxidative stress, ATP production, apoptosis and fibrosis in diabetic kidney. Our results demonstrated that PRR is localized in renal mitochondria and that this receptor expression is increased in diabetic kidney. Enhanced PRR expression in renal mitochondria contributed to development of apoptosis and fibrosis in diabetic kidney through increased intra-mitochondria oxidative stress.

## Results

### Blood glucose, body weight, 24-hour urinary albumin excretion, urinary albumin to creatinine ratio (UACR) and renal histology

**Figure 1:**
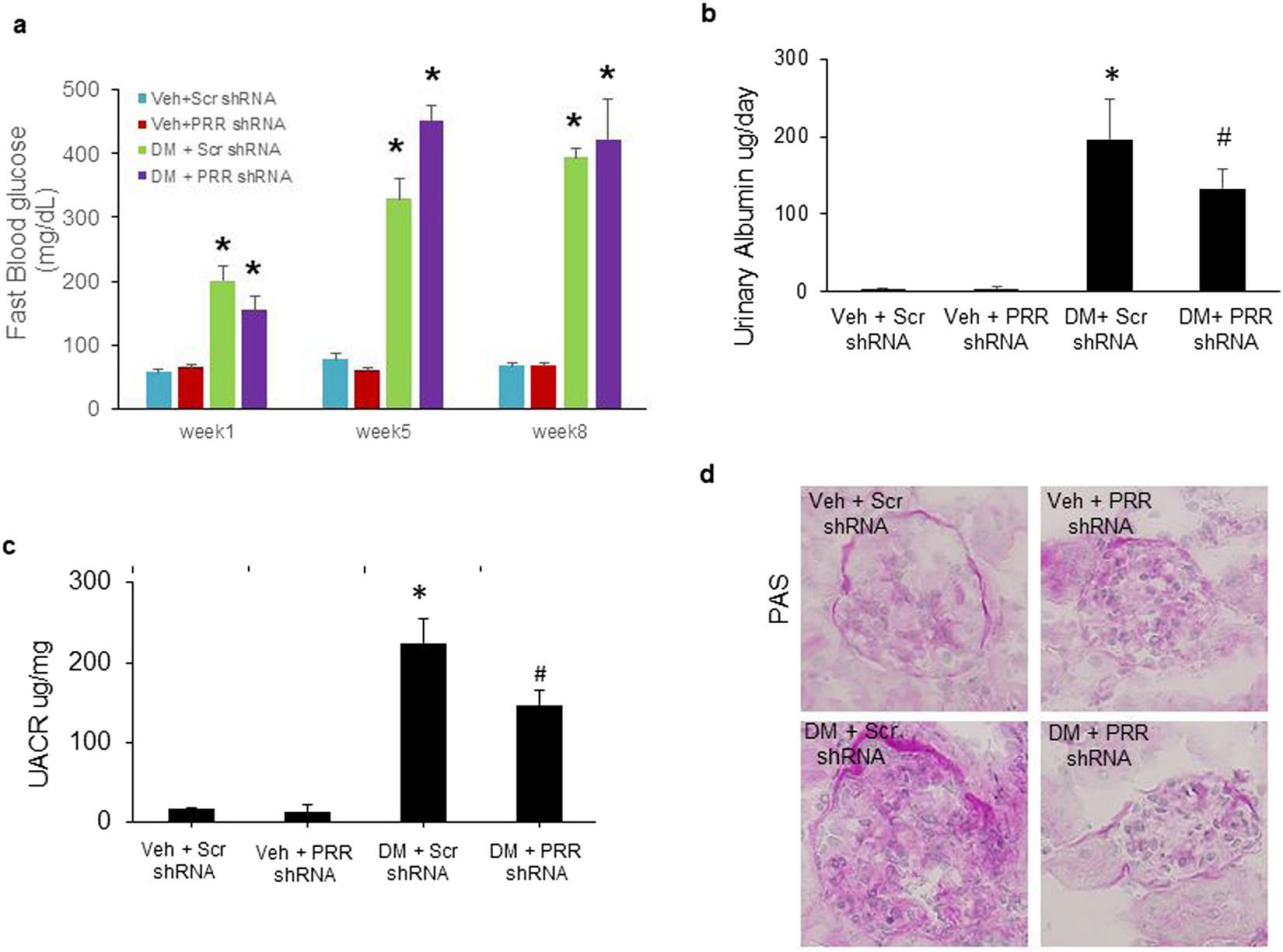
Blood glucose levels, 24-hour urinary albumin, urinary albumin to creatinine ratio (UACR), and renal PAS staining in normoglycemic and STZ-induced diabetic mice. (**a**) Fasting blood glucose levels in mice at weeks 1, 5 and 8 after diabetes induction. (n = 6–8, each group). (**b**) 24-hour urinary albumin levels in mice at the end of study (n = 6–8, each group). (**c**) UACR in mice at the end of study. (n = 6–8, each group). (**d**) Representative images showing glomerular PAS staining (dark red) in mice as indicated. PRR, (Pro)renin receptor; Veh + Scr shRNA, vehicle with scrambled PRR shRNA; Veh + PRR shRNA, vehicle with PRR shRNA; DM + Scr shRNA, diabetes with scrambled PRR shRNA; DM + PRR shRNA, diabetes with PRR shRNA. Data presented as mean ± SEM, *p < 0.05 *vs*. Veh + Scr shRNA; ^#^*p* < 0.05 *vs*. DM + Scr shRNA.

At baseline, there were no differences in blood glucose levels between groups (data not shown). Compared with normoglycemic (NG) mice, STZ-induced diabetic mice exhibited significantly higher fasting blood glucose levels (p < 0.01; Fig. 1a). Blood glucose reached the highest level at week 5 and remained elevated throughout the study period. Scrambled shRNA and PRR shRNA diabetic mice had similar levels of blood glucose. At the end of study, STZ-induced diabetes groups of mice showed a markedly increase in 24-hour urinary albumin excretion (Fig. 1b) and UACR ratio (Fig. 1c), compared to control groups. The comparison between diabetic groups of mice showed that PRR downregulation significantly reduced 24-hour urinary albumin excretion and UACR ratio. PAS staining (Fig. 1d) showed normal glomerular structure in NG mice and exhibited glomerular hypertrophy and mesangial matrix expansion in scramble shRNA diabetic mice. These histological changes were prevented in PRR shRNA diabetic mice.

### Localization of PRR to mitochondria in the kidney

**Figure 2:**
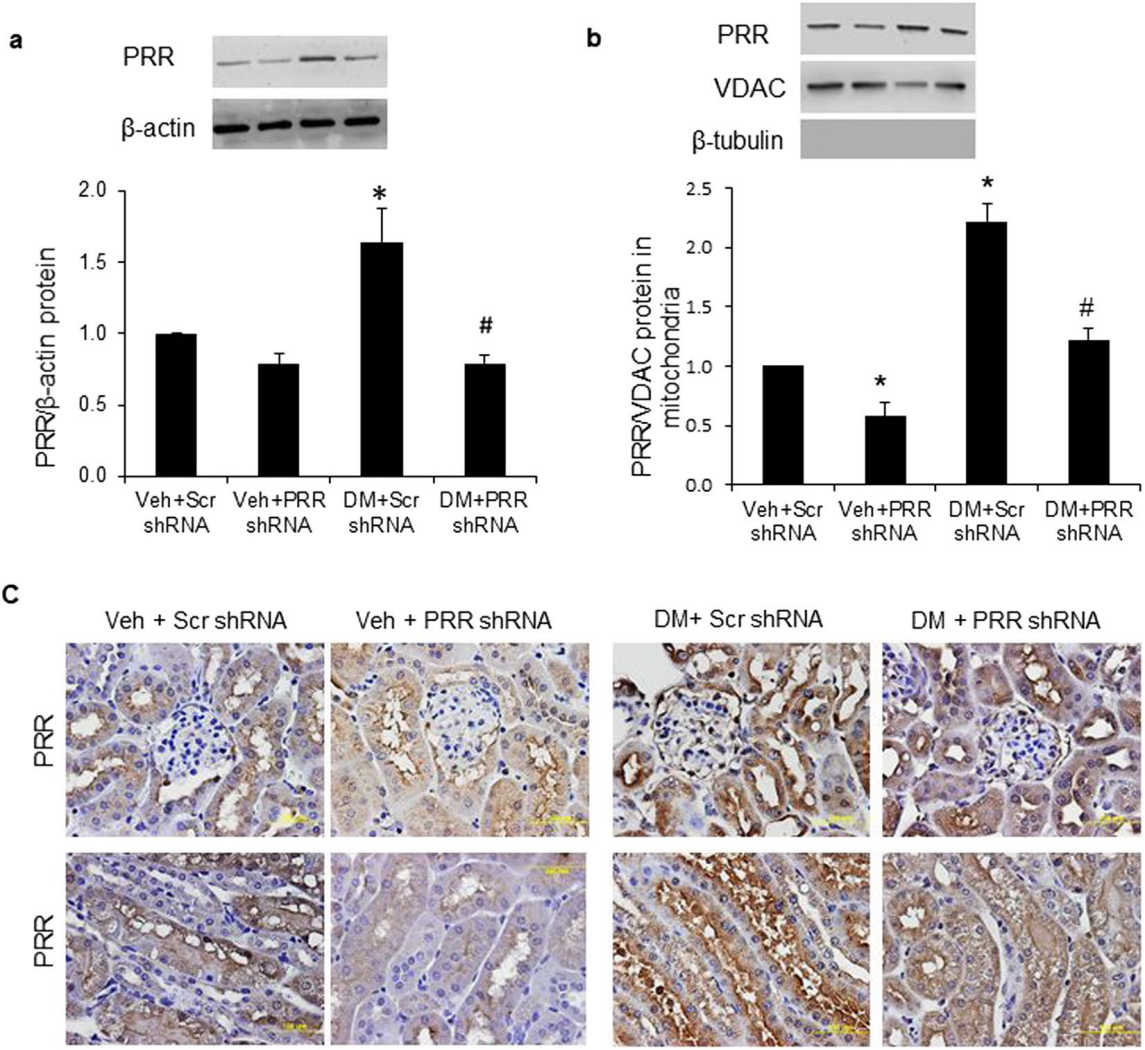
Localization of PRR in kidney and renal mitochondria of normoglycemic and STZ-induced diabetic mice. (**a**) Western blot analysis of PRR protein expression in whole kidney lysate (n = 4, each group). (**b**) Western blot analysis of PRR protein expression in renal mitochondria fraction (n = 5, each group). (**c**) Representative images of PRR immunostaining (dark brown) in mice renal cortex (top panels) and tubules (bottom panels). PRR, (Pro)renin receptor; VDAC, Voltage-dependent anion channel; Veh + Scr shRNA, vehicle with scrambled PRR shRNA; Veh + PRR shRNA, vehicle with PRR shRNA; DM + Scr shRNA, diabetes with scrambled PRR shRNA; DM + PRR shPRR, diabetes with PRR shRNA. Data presented as mean ± SEM, *p < 0.05 *vs*. Veh + Scr shRNA; ^#^*p* < 0.05 *vs*. DM + Scr shRNA.

Compared to NG mice, PRR protein expression was significantly increased in whole kidney (Fig. 2a) and renal mitochondria fraction (Fig. 2b) of scramble shRNA diabetic mice by nearly 60% and 120%, respectively. PRR shRNA did not affect PRR protein expression in whole kidney (Fig. 2a), but significantly reduced this protein expression in the renal mitochondria fraction (Fig. 2b) of NG mice, and significantly reduced PRR expression in whole kidney (Fig. 2a) and renal mitochondria fraction (Fig. 2b) of diabetic mice. Consistent with the western blot results, immunohistochemical staining showed increased PRR staining in the cortex and medulla of scramble shRNA diabetic mice (Fig. 2c). PRR immunostaining was markedly decreased in both PRR shRNA diabetic and NG groups of mice (Fig. 2c).

### Expression of NOX4 in whole kidney and renal mitochondria fraction

**Figure 3:**
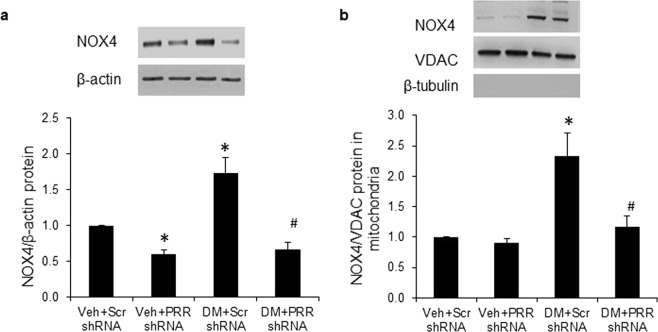
Expression of NOX4 in kidney and renal mitochondria of normoglycemic and STZ-induced diabetic mice. (**a**) Western blot analysis of NOX4 protein expression in whole kidney lysate (n = 4, each group). (**b**) Western blot analysis of NOX4 protein expression in renal mitochondria fraction (n = 5, each group). PRR, (Pro)renin receptor; VDAC, Voltage-dependent anion channel; Veh + Scr shRNA, vehicle with scrambled PRR shRNA; Veh + PRR shRNA, vehicle with PRR shRNA; DM + Scr shRNA, diabetes with scrambled shRNA; DM + PRR shRNA, diabetes with PRR shRNA. Data presented as mean ± SEM, *p < 0.05 *vs*. Veh + Scr shRNA; ^#^*p* < 0.05 *vs*. DM + Scr shRNA.

Compared to NG mice, scramble shRNA diabetic mice had significant increase in NOX4 protein expression in whole kidney (Fig. 3a) and renal mitochondria fraction (Fig. 3b) by nearly 70% and 120%, respectively. In NG mice, PRR shRNA significantly decreased NOX4 protein expression in whole kidney (Fig. 3a) but did not change this protein expression in renal mitochondria (Fig. 3b). NOX4 expression was significantly reduced in whole kidney (Fig. 3a) and renal mitochondria fraction (Fig. 3b) of PRR shRNA diabetic mice.

### SOD2 expression and activity, expression of UCP2 and ATP level in renal mitochondria

**Figure 4:**
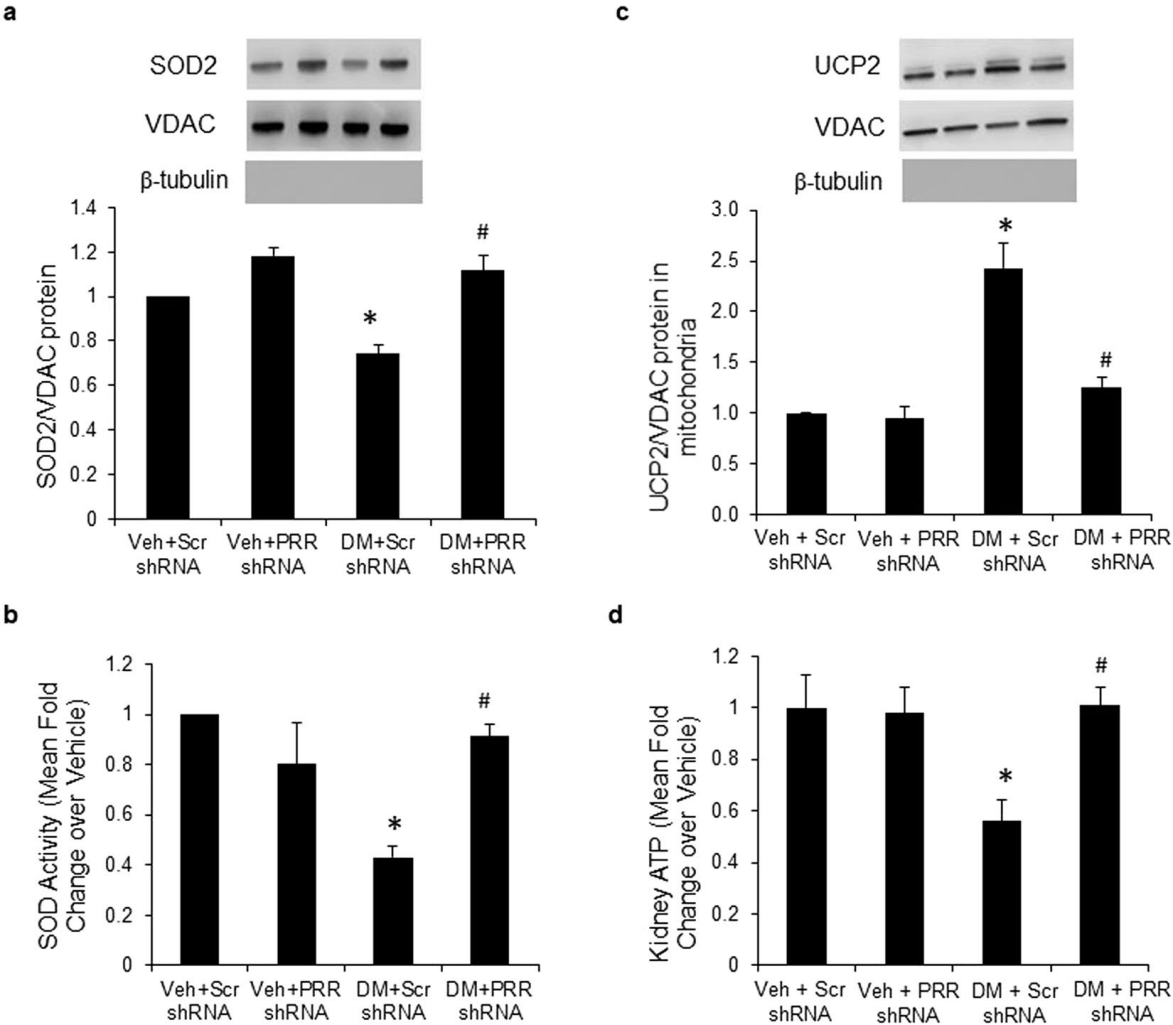
SOD2 expression and activity, expression of UCP2 and ATP levels in renal mitochondria of normoglycemic and STZ-induced diabetic mice. (**a**) Western blot analysis of SOD2 protein expression in renal mitochondria fraction (n = 4, each group). (**b**) SOD2 activity in renal mitochondria fraction. (**c**) Western blot analysis of UCP2 protein expression in renal mitochondria fraction (n = 5, each group). (**d**) ATP levels in renal mitochondria fraction. PRR, (Pro)renin receptor; Veh + Scr shRNA, vehicle with scrambled PRR shRNA; Veh + PRR shRNA, vehicle with PRR shRNA; DM + Scr shRNA, diabetes with scrambled shRNA; DM + PRR shRNA, diabetes with PRR shRNA. Data presented as mean ± SEM, *p < 0.05 *vs*. Veh + Scr shRNA; ^#^*p* < 0.05 *vs*. DM + Scr shRNA.

Both SOD2 protein expression (Fig. 4a) and activity (Fig. 4b) were significantly decreased in renal mitochondria of scramble shRNA diabetic mice by nearly 30% and 60%, respectively. These changes were reversed in PRR shRNA mice. UCP2 protein expression (Fig. 4c) was significantly increased by nearly130% in renal mitochondria of scramble shRNA diabetic mice. The UCP2 overexpression was inhibited in PRR shRNA diabetic mice (Fig. 4c). ATP levels (Fig. 4d) were significantly decreased by nearly 50% in renal mitochondria of scramble shRNA diabetic mice. PRR shRNA reversed the decrease in ATP levels in diabetic mice. In NG mice, PRR shRNA did not promote any changes in SOD2 expression and activity, UCP2 expression, or ATP levels.

### Renal expressions of Sirt1, Forkhead Box O (FOXO3a) and Caspase-3

**Figure 5:**
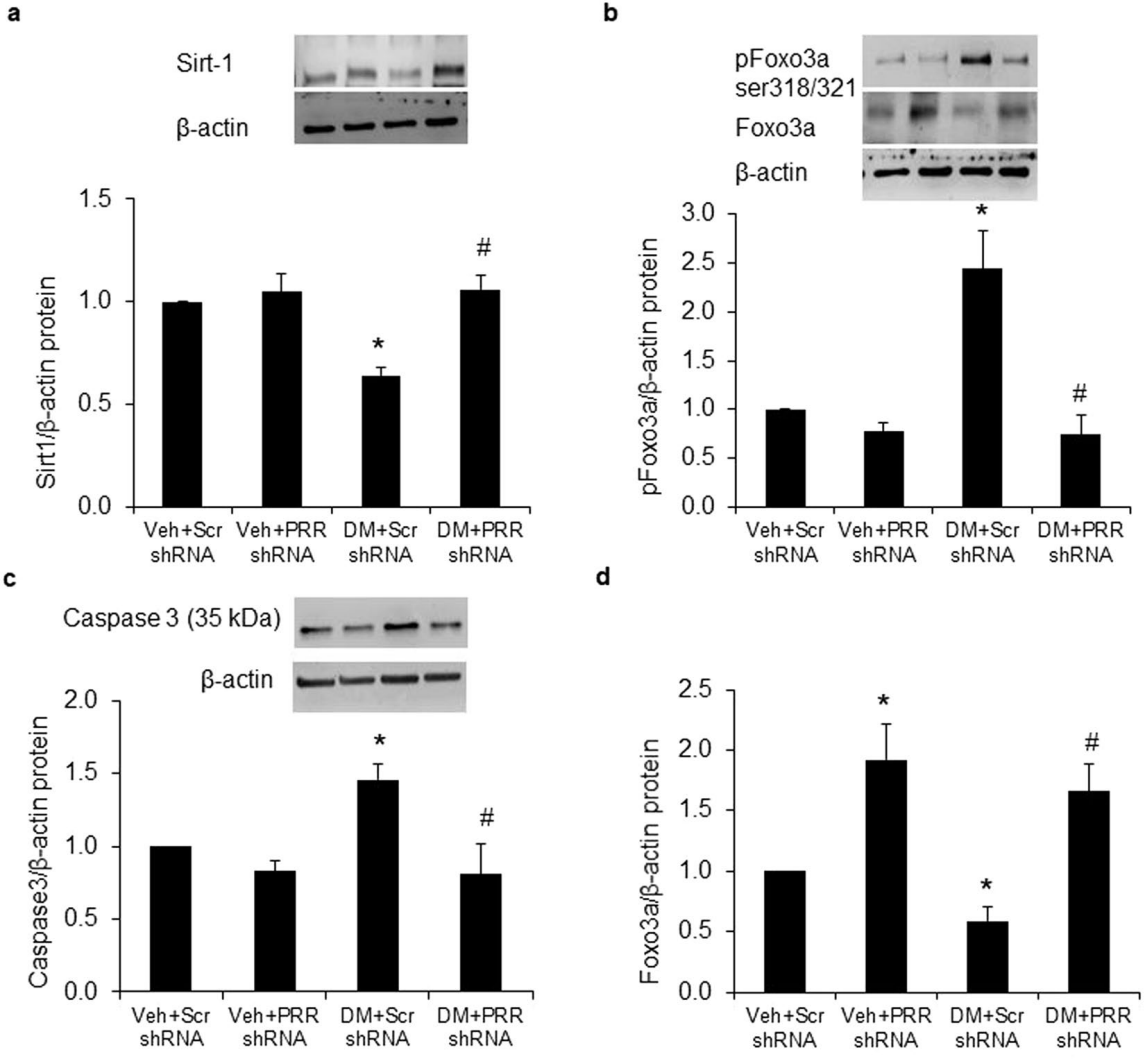
Expression of Sirt1, FOXO3a and Caspase-3 in kidney of normoglycemic and STZ-induced diabetic mice. (**a**) Western blot analysis of renal Sirt1 protein expression (n = 4, each group). (**b**) Western blot analysis of renal pFOXO3a ser318/321 and total-FOXO3a protein expression (n = 4, each group) (**c**) Western blot analysis of renal Caspase 3 protein expression (n = 5, each group). PRR, (Pro)renin receptor; Veh + Scr shRNA, vehicle with scrambled PRR shRNA; Veh + PRR shRNA, vehicle with PRR shRNA; DM + Scr shRNA, diabetes with scrambled shRNA; DM + PRR shRNA, diabetes with PRR shRNA. Data presented as mean ± SEM, *p < 0.05 *vs*. Veh + Scr shRNA; ^#^*p* < 0.05 *vs*. DM + Scr shRNA.

Compared with NG mice, protein expressions of Sirt1 (Fig. 5a) and total-FOXO3a (Fig. 5d) were both significantly decreased by 40%, while protein expressions of p-FOXO3a ser318/321 (Fig. 5b) and Caspase3 (Fig. 5c) were increased by nearly 140% and 50% in kidney of scramble shRNA diabetic mice. PRR shRNA significantly reversed all these protein expressions in diabetic kidney. In NG mice, PRR shRNA significantly enhanced renal total-FOXO3a expression by 80%, but did not affect the expressions of Sirt1, phospho-FOXO3a ser318/321 or Caspase3.

### Immunostaining of collagen IV and fibronectin in the kidney

**Figure 6:**
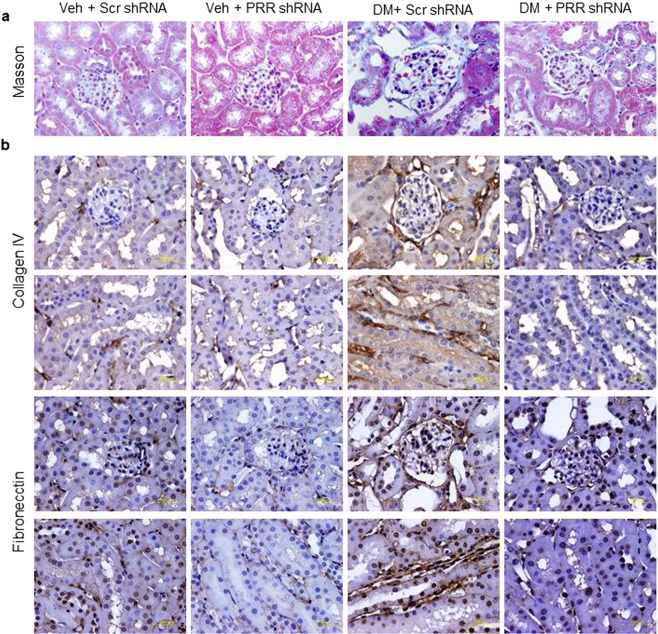
Masson staining and immunostaining of collagen IV and fibronectin in kidney of normoglycemic and STZ-induced diabetic mice. (**a**) Masson’s trichrome stain deposition in mice renal cortex. (**b**) Immunohistochemical staining deposition of collagen IV (dark brown; top two panels) and fibronectin (dark brown; lower two panels) in mice renal cortex and tubules. PRR, (Pro)renin receptor; Veh + Scr, vehicle with scrambled PRR shRNA; Veh + shPRR, vehicle with PRR shRNA; DM + Scr, diabetes with scrambled PRR shRNA; DM + shPRR, diabetes with PRR shRNA.

Compared to NG mice, the Masson’s trichrome staining analysis showed accumulation of collagen in the kidneys of STZ-induced diabetes groups (Fig. 6a). Similarly, diabetic mice showed an increased intensity of immunostaining for collagen IV and fibronectin (Fig. 6b) in renal glomeruli and tubules, which was predominantly localized to basement membranes, indicating the presence of interstitial fibrosis. PRR shRNA partially prevented the renal accumulation of collagen IV and fibronectin (Fig. 6b).

### Renal expressions of NF-κB, collagen IV and fibronectin

**Figure 7:**
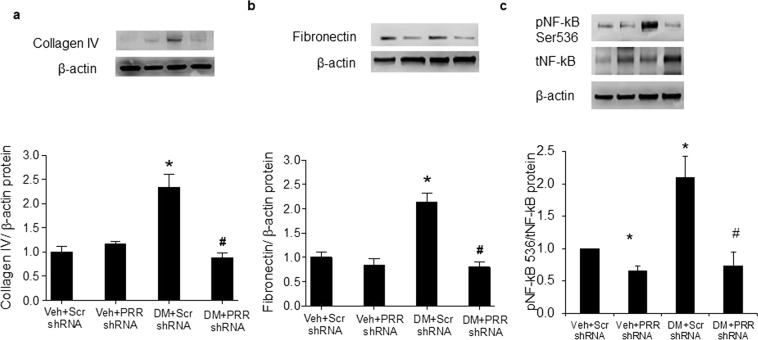
Expression of NF-κB, collagen IV and fibronectin in kidney of normoglycemic and STZ-induced diabetic mice. (**a**) Western blot analysis of renal collagen IV protein expression (n = 4, each group). (**b**) Western blot analysis of renal fibronectin protein expression (n = 5, each group). (**c**) Western blot analysis of renal pNF-κB ser536 and total- NF-κB protein expression (n = 4, each group). PRR, (Pro)renin receptor; Veh + Scr shRNA, vehicle with scrambled PRR shRNA; Veh + PRR shRNA, vehicle with PRR shRNA; DM + Scr shRNA, diabetes with scrambled shRNA; DM + PRR shRNA, diabetes with PRR shRNA. Data presented as mean ± SEM, *p < 0.05 *vs*. Veh + Scr shRNA; ^#^*p* < 0.05 *vs*. DM + Scr shRNA.

Consistent with the immunostaining results, both renal collagen IV (Fig. 7a) and fibronectin (Fig. 7b) proteins were significantly increased in scramble shRNA STZ-induced diabetes mice. In contrast, collagen IV and fibronectin were significantly reduced in PRR shRNA diabetic mice. PRR shRNA did not change proteins expression of collagen IV and fibronectin in NG mice. In addition, scramble shRNA diabetic mice exhibited significant increase in p-NF-κB level by about 110% compared with NG mice while PRR shRNA significantly decreased p-NF-κB levels in NG and diabetic mice by about 30% and 60%, respectively.

## Discussion

Oxidative stress plays a critical role in the pathogenesis of diabetic complications, including the renal and cardiovascular systems^24,25^. Mitochondria is a major source of ROS within most mammalian cells^26–28^. Increased mitochondria oxidative stress in response to hyperglycemia is associated with mitochondria dysfunction and damage to renal structure and function^29–31^.

In the present study, we hypothesized that PRR localized in renal mitochondria contributes to development of renal fibrosis and apoptosis through oxidative stress-induced mitochondria dysfunction. To our knowledge, this is the first report demonstrating that PRR mediates the renal mitochondria ROS production and renal damage through hyperglycemia. First, we demonstrated that PRR is localized in the renal mitochondria and that this protein expression was upregulated in both total kidney tissue and renal mitochondria fraction in response to hyperglycemia. Then, we demonstrated that increased PRR expression in renal mitochondria was associated with enhanced NOX4/UCP2 production and reduced SOD2 activity and ATP synthesis. Collectively, these changes were accompanied by increased apoptosis, inflammation, and fibrosis in diabetic kidney. Finally, we showed that down-regulation of PRR reversed hyperglycemia-induced mitochondria dysfunction, renal inflammation, apoptosis and fibrosis in diabetic kidney. These results suggest that in diabetes, upregulation of renal PRR contributes to renal mitochondria dysfunction and development of renal inflammation, apoptosis and fibrosis.

Previous studies reported that high glucose increased PRR expression and that this receptor activity plays a major role in development of diabetic kidney disease^3,4,9^. PRR is highly expressed in renal cells including podocytes^8^, mesangial cells^3^, endothelial cells^32^, vascular smooth muscle cells^33,34^, and collecting duct cells^35,36^. PRR was localized to subcellular components of different cell types such as the cytomembrane^37^ and some organelles such as endoplasmic reticulum^37^, lysosome and Golgi^38^. In the present study, we report for the first time the localization of PRR in mitochondria of kidney tissue and the increase of its expression in presence of diabetes.

Oxidative stress is increased in diabetes and contributes to the progression of its complications. NOX4 is a member of the NADPH oxidase family and it is localized to the inner mitochondria membrane or to the interior of the outer membrane in various renal and endothelial cell types^39^. Upregulation of NOX4 was previously demonstrated to promote oxidative stress and to induce mitochondria dysfunction and apoptosis in cardiac myocytes^40^ and renal cells^39^. Our study demonstrated that increased expression and activity of renal mitochondria NOX4 in control diabetic mice were reversed by downregulation of PRR expression. This is a novel finding suggesting that increased PRR contributes to enhanced mitochondria NOX4 expression and activity in diabetic kidney disease.

In addition, superoxide dismutase provides the first line of defense against ROS mediated cell injury by catalyzing the proportion of superoxide anion to be converted to molecular oxygen and peroxide^13,41^. SOD2 is mainly localized in mitochondria and acts by transforming toxic superoxide, a byproduct of the mitochondria electron transport chain, into hydrogen peroxide and diatomic oxygen, to clear mitochondria ROS and confer protection against cell death^42^. Lower levels of SOD2 leads to increased expression of uncoupling proteins^43,44^. UCPs are members of a large family of mitochondria anion carrier proteins and causes mitochondria proton leaking^18,45^, which separates oxidative phosphorylation from ATP synthesis leading to energy dissipation as heat and decreased mitochondria ATP synthesis^46^. In the present study, we demonstrated that hyperglycemia caused reduction of renal mitochondria SOD2 and enhancement of UCP2, then reduced ATP production by the mitochondria. We also hypothesized that hyperglycemia-induced mitochondria dysfunction is mediated by enhanced activity of PRR/NOX4 signaling pathway. Our results demonstrated that downregulation of renal mitochondria PRR via PRR shRNA delivery efficiently blocked hyperglycemia-induced dysfunction of mitochondria by reversing SOD2 levels, inhibiting UCP2 protein levels, and increasing ATP synthesis. It is reported that UCP2 may function as a sensor and negative regulator of mitochondria ROS production in response to hyperglycemia^15,47^. In our study, PRR shRNA decreased the UCP2 level to normal level in diabetic mice and kept the antioxidative stress ability of UCP2. These data suggest that renal mitochondria PRR is upstream of this signaling pathway. To our knowledge, this is the first report showing that renal mitochondria PRR mediates hyperglycemia-induced dysfunction of mitochondria.

ATP is a key molecule of cellular regulation and maintenance of ion homeostasis and apoptosis^48^. Inhibition of mitochondria ATP synthesis plays an important role in stimulating apoptosis^48,49^. In addition, caspase proteins are also crucial mediators of apoptosis. Caspase-3 activates death protease and is responsible for chromatin condensation and DNA fragmentation^49,50^. Our previous studies showed that high glucose enhanced caspase-3/7 activity and increased the number of apoptotic cells in podocytes, and also that downregulation of PRR reversed the high-glucose-induced cell death^4^. In the present study, we confirmed our previous findings that hyperglycemia increased renal caspase-3 expression, and furthermore that knockdown of PRR in mitochondria inhibited the expression of caspase-3. These results suggested that mitochondria PRR plays a key role in hyperglycemia-induced apoptosis in kidney cells.

ROS as well as other stressful stimuli can modulate FOXO activity through posttranslational phosphorylation and acetylation^51^. FOXO proteins are a family of transcription factors that can be inhibited by the deacetylase sirtuin (Sirt1)^52,53^. Activation of Sirt1 can prevent the hyperglycemia-induced vascular cell dysfunction in mice^54^. Sirt1 binds to and deacetylate FOXO3a, thus increasing FOXO3a ubiquitination and protecting against oxidative stress-induced apoptosis^55^. FOXO3a also has been shown to play a role in the enhancement of autophagy by transactivation of autophagy-related genes. Phosphorylation of FOXO3a at serine residues 318 and 321 can destabilize FOXO3a, decreasing expression of autophagy genes and limiting autophagic recycling of nutrients^56^. Our previous studies showed that PRR mediated high glucose induced reduction in autophagy function and increased cell apoptosis in podocytes^4^. In the present study, we demonstrated that hyperglycemia decreased Sirt1, total FOXO3a protein levels and phos-FOXO3a Ser318/321. These effects were reversed by downregulation of PRR, suggesting that PRR can accelerate hyperglycemia induced apoptosis by reduction of Sirt1/FOXO3a signaling pathway.

Hyperglycemia-induced mitochondria injury also contributed to development of renal inflammation^57^. Renal inflammation plays a central role in the initiation and progression of fibrosis in chronic kidney disease^58,59^. NF-κB triggers the inflammatory processes by activating large number of pro-inflammatory genes^60–62^. Previous studies demonstrated that diabetes was associated with increased deposition of collagen IV and fibronectin^62–64^. In our study, collagen type IV and fibronectin, well-characterized markers of tissue fibrosis, were predominantly localized to basement membranes of the glomerulus, proximal and distal tubules in diabetic mice. Our data also showed that diabetes increased renal NF-κB ser536 expression suggesting activation of renal inflammatory process. Downregulation of PRR decreased hyperglycemia-induced activation of renal NF-κB and protein expression of fibronectin and collagen IV. These results confirm the involvement of PRR in mediating hyperglycemia-induced renal inflammation and fibrosis.

Further studies should investigate the evidence whether the mitochondria PRR has a role that is separate from its role at other cell sites, whether it functions independently and where does it originate. In summary, we provide the first evidence for PRR localization in the mitochondria and its role in mitochondria dysfunction, inflammation, apoptosis and fibrosis in diabetic kidney. We conclude that NOX4/UCP2/NF-κB and Sirt1/FOXO3a signaling pathways mediate the renal effects of PRR in diabetes. This finding may serve as a potential therapeutic tool in the management of diabetic kidney disease.

## Materials and Methods

### Animals

The University of Virginia Animal Care and Use Committee approved all study protocols. All methods were performed in accordance with the guidelines and regulations of the University of Virginia Animal Care and Use Committee and the National Institutes of Health. Eight-week-old male C57BL/6 (BL6) mice were purchased from Jackson Laboratory and kept in a 12-hour dark/light cycle and were provided standard chow and tap water ad libitum. Mice were randomly divided into four treatment groups: normoglycemic (control) groups injected with either PRR ScrRNA (Veh + PRR ScrRNA, n = 7) or PRR shRNA (Veh + PRR shRNA , n = 8), and STZ induced diabetic mellitus (DM) groups injected with either PRR ScrRNA (DM + PRR ScrRNA, n = 8) or PRR shRNA (DM + PRR shRNA, n = 10). To induce PRR shRNA into the mice kidney, all mice underwent left uninephrectomy prior to diabetes induction as we previously described^65^. In brief, 50 µl PRR shRNA or PRR ScrRNA (Viral vector core, University of Iowa, Iowa City, IA) were directly microinjected into the right kidney of the mice using a minipump at 3 µl/min.

Thereafter, diabetes was induced by intraperitoneal administration of 55 mg/kg of streptozotocin (STZ; Sigma-Aldrich, Saint Louis, MO) for 5 consecutive days. Normoglycemic control mice were treated with an equal volume of vehicle (0.9% NaCl). Body weight was measured at baseline and at the end of study. Mice were fasted overnight at weeks 1, 5 and 8 to collect blood from tail vein and blood glucose were determined using glucometer. At the end of study, mice were sacrificed and kidneys were harvested for mitochondria isolation, protein extraction, immunostaining and morphological examinations.

### 24-h urinary albumin and urine albumin-to-creatinine ratio (UACR) analysis

For urine collections, mice were placed in individual metabolic cages for a period of 24-h in the last week of study and urine samples were collected and kept at −80 °C until assayed. Urinary albumin was determined by using a commercial mice albumin ELISA kit (Exocell, Philadelphia, PA), and urine creatinine was assessed using creatinine assay kit (Cayman Chemical, Ann Arbor, MI). UACR was presented in micrograms per milligram and used as a marker for development of diabetic kidney disease.

### Renal mitochondria isolation

Commercially available mitochondria isolation kit for tissue (Abcam, ab110169, Cambridge, MA) was used to isolate mitochondria from the kidney tissue according to manufacturer instructions. The mitochondrial preparation followed cell rupturing, low speed centrifugation to remove large particles and finally centrifugation at high speed to isolate mitochondria. In brief, 20 µg of mouse kidney tissue was washed twice with wash buffer and minced in pre-chilled dounce homogenizer with isolation buffer. Homogenate was centrifuged at 1,000 g for 10 mins at 4 °C, the supernatant was then mixed with isolation buffer and centrifuged at 12,000 g for 15 mins at 4 °C, pellet was resuspended in 100 µl of isolation buffer supplemented with protease inhibitor cocktail and aliquots were stored at −80 °C until use.

### Mitochondria purity assessment

The integrity and purity of the mitochondria fraction were confirmed by western blot analysis for several protein markers. Twenty micrograms of protein were separated by 4–12% gradient SDS-PAGE, transferred onto a PVDF membrane, electrotransferred to polyvinylidene difluoride membrane (PVDF) (Millipore, Billerica, MA), and probed with primary antibody followed by incubation with horseradish peroxidase-labeled IgG (1:5000) depending on the primary antibody. The purity of the mitochondria fraction was tested by screening for mitochondria protein utilizing the following antibodies: anti-VDAC (1:1000, 48661, cell signaling, CST), anti-β -tubulin (1:5000, ab21058, Abcam) for the cytosol, anti-calnexin for the nuclear and endoplasmic reticulum (2433 S, cell signaling, CST) in the isolated mitochondria versus in the supernatant fraction. We found that all mitochondria preparations were essentially free of cytosolic and nuclear contaminants. Together, these results attest to the purity of our mitochondria fractions, i.e., they were substantially free of cytosolic, nuclear or endoplasmic reticulum components.

### Renal mitochondria SOD2 activity measurement

SOD2 activity was detected by using a commercially available superoxide dismutase assay kit (706002, Cayman Chemical, MI) according to manufacturer instructions. Kidney tissue samples were rinsed with PBS and homogenized in cold 20 mM HEPES buffer, centrifuged at 1,500 g for 5 min at 4 °C, supernatant was collected and used for assay. In brief, 10 µl of tissue samples or standard were added into each well of 96 well plate followed with 200 µl of the diluted radical detector. The reaction was initiated by adding 20 µl of Xanthine Oxidase into each well, plate was incubated on a shaker for 30 minutes at room temperature. Absorbance was measured at 440 nm using a plate reader.

### Renal mitochondria ATP levels measurement

Renal mitochondria ATP levels were measured by using a commercially available ATP assay kit (K354-100, Biovision, CA) according to manufacturer instructions. Kidney tissue samples were homogenized in ATP assay buffer, the homogenates were added into 96-well plate. 50 µl of the reaction mix was added to each well containing the ATP standard and test samples. After 30 min incubation, absorbance was measured at OD 570 nm on microplate reader (Epoch, BioTek, Japan). ATP concentrations were calculated by construction of ATP calibration curve.

### Western blot analysis

Western blot analysis was performed as described previously^8^. In brief, whole kidney homogenates were lysed in the presence of protease inhibitors. Clear protein extracts were obtained by centrifugation at 12,000 g for 10 min. 20 μg proteins were transferred to subjected to 4%-12% gradient SDS-PAGE, transferred onto a PVDF membrane filters (Bio Rad). PVDF membranes were blocked with 5% dry milk for 1 h. Membranes were incubated in the primary overnight at 4 °C. The following antibodies used in the study are: anti-PRR (1:1000, HPA003156, Sigma), anti-NOX4 (1:1000, ab133303, Abcam), anti-SOD2 (1:1000, ab13533, Abcam), anti-UCP2 (1:1000, ab203244, Abcam), anti-Caspase 3 (1:1000, CST #9665, Cell signaling), anti-pNF-κB Ser536 (1:1000, ab86299, Abcam), anti- tNF-κB (1:1000, ab16502, Abcam), anti-collagen IV (1:1000, ab6586; Abcam), anti-fibronectin (1:1000, ab2413; Abcam), anti-sirt1 (1:200, sc-15404, Santa Cruz), anti-pFOXO3a ser318/312 (1:1000, CST #9465, Cell signaling), anit-FOXO3a (1:200, sc-48348, Santa Cruz), and anti-β-actin (1:5000, sc-47778, Santa Cruz). This was followed by incubation with the corresponding secondary antibody (horseradish peroxidase-labeled IgG, 1:1000). β-actin was used to normalize per total amount of loaded proteins. Chemiluminescence blot images were captured by the UVP imaging and analyzed by using Image J software (NIH, Bethesda, MD).

### Histology staining in kidney

Sections (4-µm thick) cut from 4% formalin-fixed, paraffin-embedded kidney samples were used for periodic acid-Schiff (PAS) staining and Masson’s trichrome staining.

### Immunohistochemical staining

Immunohistochemical staining was performed to determine renal cellular expression of PRR (1:100, Sigma Aldrich HPA003156), collagen IV (1:200, Abcam ab6586), and fibronectin (1:400, Abcam ab2413). 4-µm-thick kidney sections were used. Heat-induced antigen retrieval was conducted in 10 mM sodium citrate (pH 6.0). Endogenous peroxide activity was suppressed by 3% peroxide-methanol solution. VECTASTAIN® ABC KIT (Vector Laboratories, Burlingame, CA) was used for blocking and color reaction. Immunostaining was performed by incubation with primary antibody overnight at 4 °C followed by 30 min of incubation with a secondary antibody conjugated with biotin at room temperature.

### Statistical analysis

Data analysis was carried out using STATISTICA version 5.0 (StatSoft, Tulsa OK). Values are expressed as mean ± SEM. Significant differences among multiple groups were examined using two-way analysis of variance (ANOVA) with repeated measures, and the *Bonferroni* correction method as a *post-hoc* test. P < 0.05 was considered statistically significant.
